# On Some Diseases Occurring in Men Who Worked in a Sewer
1Read at the Meeting of the Bristol Medico-Chirurgical Society on November 10th, 1897.


**Published:** 1898-03

**Authors:** F. H. Edgeworth

**Affiliations:** Assistant-Physician to the Bristol Royal Infirmary


### ON SOME DISEASES OCCURRING IN MEN WHO WORKED IN A SEWER.[1]

BY

F. H. Edgeworth, M.B., B.A. Cantab., B.Sc. Lond.,

*Assistant-Physician to the Bristol Royal Infirmary.*

During the months of July and August last those who lived or passed by St. John's Road, Clifton, were only too conscious that the sewer there was being repaired.

Of the men engaged in the work who fell ill, some were admitted to the Royal Infirmary under my care; I have obtained notes of others from the doctors by whom they were treated, and have thought that a few remarks on the conditions observed may be of interest to the Society, more especially as some of my colleagues and friends have had cases of illness in their practice which were apparently due to the same cause.

The Clifton high-level sewer runs along Alma Road, at a considerable depth underground, across the College Close, to the top of the New Zigzag, and discharges its contents into the Avon at the bottom.

The main sewer receives the smaller one, which runs down St. John's Road. This was found not to be large enough in the lower part of its course, that is to say, in that part of St. John's Road which runs obliquely into Alma Road. So the old drain, which is from sixteen to twenty-five feet underground and one

[1] Read at the Meeting of the Bristol Medico-Chirurgical Society on November 10th, 1897.

foot nine inches by one foot three inches in diameter, was replaced by a new one measuring three feet by two feet. A channel was first excavated, partly by blasting especially in the lower part of the road, partly by removal of made ground. The excavation took place below and to one side of the old sewer, in sewage-soaked ground, for the old bricks were hand-made and somewhat porous, with not very closely-fitting joints. The new sewer was then built in the channel thus made, and the two ends of the old one stopped up.

Now, it is interesting to observe that the excavating and building were carried out by two distinct sets of men—miners and bricklayers; and whilst not one of the four bricklayers was affected, every-one of the eight miners suffered in a greater or less degree. Of these eight miners two had no definite symptoms and did not come under medical observation, though I am told that their appetites failed and they looked ill. The other six were affected more seriously. One had gastric symptoms, one had gastro-intestinal catarrh, two others had gastro-intestinal catarrh followed by jaundice, one had an anomalous form of typhoid fever, and the last had pneumonia followed by jaundice.

The men who had symptoms of gastric or gastro-intestinal catarrh were ill during a few days only, and the cases presented no unusual features. One of the two who suffered from gastro-intestinal catarrh followed by jaundice was under my care in the Royal Infirmary. The gastro-intestinal symptoms, which were ushered in by vomiting and diarrhœa, general pains and fever, subsided within a week, and he was admitted on the tenth day of his illness, complaining of weakness and jaundice only. The jaundice was of moderate degree, the conjunctivæ and skin being of an orange tint. Bile pigments and a trace of albumen were present in the urine. The thoracic and abdominal organs were normal, as far as physical examination went, and neither the spleen nor the liver was enlarged. It is of importance to note that the motions were bile-stained. Considerable muscular weakness and debility were present. The most remarkable feature in the case was that fever was present, the temperature varying between 99° and 100.6° for eleven days.

As it gradually subsided the jaundice grew less, and the bile pigments and albumen disappeared from the urine. The patient gradually recovered and went home in about three weeks. In the other case of gastro-intestinal catarrh followed by jaundice, the symptoms were at the onset similar to those of the one just related, but the jaundice which followed was accompanied by slate-coloured stools, and the fever did not last longer than the intestinal symptoms.

The fifth case was one of typhoid fever; it began suddenly with general pains and a high temperature, which lasted for a week or ten days, to relapse two days later. And with this relapse a rash very like a typhoid one developed; from this time the patient, however, gradually recovered. No certificate of typhoid fever was sent to the Sanitary Authority, as no certain diagnosis was arrived at, but it was thought that in all probability it was an anomalous or disguised typhoid fever. Since then, some two and a-half months after the illness, Dr. Davies kindly tested the patient's blood for me by Widal's reaction. The blood serum was found to give the characteristic clumping in 2½ hours when diluted fifteen times. Taking into consideration the clinical symptoms, and the positive result of Widal's test, it may with some safety be inferred that the man suffered from an anomalous form of typhoid fever.

The sixth and last patient was first taken ill with pneumonia of the base of the right lung, and during convalescence from this he became jaundiced, and but very slowly recovered.

Of the six men, then, who had some definite illness, four had symptoms of either gastric or gastro-intestinal catarrh, and two of these subsequently developed jaundice, one had pneumonia followed by jaundice, and one had typhoid fever.

One of the cases of jaundice following gastro-intestinal catarrh presented somewhat unusual features; viz., its association with fever and absence of evidence of any block in the common bile-duct. Jaundice of itself does not give rise to fever, nor is a heightened temperature present in ordinary so-called catarrhal jaundice. These phenomena point to the supposition that the fever and jaundice were collateral results of the same cause, that they were toxæmic in origin; and this supposition is strengthened by the fact that concurrently with the symptoms albumen was present in the urine. The late Sir George Johnson showed that sewer-gas poisoning will cause nephritis, and neither was the fever high enough nor the jaundice long enough continued to give rise to albuminuria of itself.

The jaundice which followed the other case of gastro-intestinal catarrh was probably due to some obstruction in the common bile-duct, for the motions were not clay-coloured and, unlike the case just spoken of, the fever did not last longer than the intestinal symptoms.

The fact that jaundice was a sequela of the case of pneumonia is of some interest, for this complication, which occurs in about seven per cent. of cases, is not due to obstruction of the common bile-duct, but is of toxæmic origin. As far as our present knowledge goes, jaundice following pneumonia does not point to a sewer-gas origin, but it is perhaps a little suggestive that one of the two miners who worked in St. John's Road and had no definite illness, suffered a few years ago after working in an especially foul sewer from pneumonia followed by jaundice.

The case of typhoid fever is of especial interest from a clinical point of view, in that the phenomena did not allow of a positive opinion. It is a case in which most valuable evidence was obtained by the application of Widal's test, evidence which clinched the diagnosis.

It is not for me to enter upon the general question of the value and limits in time of Widal's reaction as a test for typhoid fever, but I would remark that a positive result has been found by Tyson in 98 per cent. of cases in which the diagnosis of typhoid fever was arrived at from clinical evidence. And the following striking illustration of the utility of the test may be adduced: In the latter part of July last four children of one family were admitted to the Royal Infirmary. Three undoubtedly had typhoid fever, one having intestinal hemorrhage, and the other two were operated on for perforating intestinal ulcers. All these three gave Widal's reaction. The fourth child, under the care of Dr. Prowse, and afterwards when convalescent, of myself, had a fever which lasted only one week with no symptoms other than that which could be put down to the fever.

Her blood, examined by Dr. Davies, gave a most decided reaction to the test. Such a case, to my mind, establishes the old doctrine that occasionally typhoid fever may be, as used to be said, abortive—running a course of much less than the typical three or more weeks.

Cases of sewer-gas poisoning can be divided, as is well known, into two categories,—those of sudden onset due to asphyxia from sulphuretted hydrogen or deficiency of oxygen, or from both of these causes, and those cases of illness which in all probability are due to infection by micro-organisms of various kinds in sewage. The cases described obviously fall into the latter group.

Work in sewers is not such an unhealthy occupation as might at first sight be supposed, for the air in sewers where the sewage is in movement is not so very impure, and one knows clinically that it is rare for sewer men to come under medical observation for any illness which might in any way be attributed to the nature of their employment.

But if sewage be stagnant and decomposing there is an enormous production of micro-organisms, which may infect the neighbouring atmosphere and be a fruitful cause of disease. And in the particular cases in question it is to be remarked that men, who were accustomed to work in sewers, were taken ill whilst excavating ground which was soaked with decomposing sewage and gave off a most offensive smell. The occurrence presents then no deviation from the usual type. Illness due to sewer-gas poisoning may be of various kinds and severity: general ill-health without any obvious lesion, feverish attacks, tonsillitis, gastro-intestinal affections, and nephritis, are no doubt the commonest. In these workmen it is of interest to note that no case of tonsillitis occurred; though I have been told of a case which arose close to St. John's Road at the time, and which was probably of sewer-gas origin.

The case of toxæmic jaundice following gastro-intestinal catarrh is worthy of note in that it suggests first that jaundice following this complaint may sometimes be of toxæmic origin, that both features are collateral results of one cause, and also that simple jaundice not following gastro-intestinal catarrh, for which we can so often find no obvious cause, may in a few cases be of toxic and probably of sewer-gas origin. Similarly, if jaundice follow pneumonia, such an origin may, though with little probability of finding it, be at any rate looked for.

None of the family of the man who developed typhoid fever suffered in any way, though he has many children. The occurrence then suggests, whilst it does not prove, that the old sewage was not merely stagnant and decomposing, but also specifically infected with typhoid bacilli.

In conclusion, I must offer my best thanks to Drs. Ormerod, Stevens, Stephenson, and Burroughs for having so kindly given me details of the cases which came under their care.

---

In the discussion which followed Dr. Edgeworth's paper, Mr. Paul Bush mentioned that while the sewer was open he had three cases in Alma Road, all having a temperature of 102° to 103° for seven to ten days, severe form of ulcerated throat in two patients, one had jaundice, and one had albumen in the urine. The three cases occurred in one house, all commenced with gastro-intestinal symptoms; he did not look upon any of them as cases of diphtheria or enteric fever.—Mr. Munro Smith spoke of various cases of illness in the neighbourhood, chiefly taking the form of gastro-intestinal irritation, headaches, sore throats, and rise of temperature.—Mr. Hancocke Wathen remarked that, it is a well-known fact that sore throat, and such allied troubles, are associated with sewer-gas poison. But that a given case was typhoid caused by this sewer-gas did not necessarily need a previous case of that disease. The origination of typhoid *de novo* was held by Murchison, Jenner, and Dyke of Merthyr, and he was quite willing to follow such leading.—Dr. D. S. Davies commented on the absence from Dr. Edgeworth's cases of any diphtheria or suspected diphtheria; a result which experience would lead us to expect, but which is much opposed to popular opinion, which generally looks upon sewers and diphtheria as cause and effect.—Dr. B. M. H. Rogers was of opinion that the infection came more from the disturbance of the saturated soil rather than the smells themselves, for Dr. Haldane had shown that a drain, even in a large city, was in reality a healthy place and remarkably free from micro-organisms.—Dr. A. J. Harrison considered that the cases described arose from sewage-contaminated ground, and were shown to be amongst men who were navvies and not hardened by practice.—Mr. J. C. Heaven was of opinion that the cases mentioned by Dr. Edgeworth were due to the disturbance of sewage-saturated soil rather than to the opening of the sewer itself, and were caused more probably by products cf decomposed animal matter, in the form of ptomaines and leucomaines, than by germs, which are generally accepted not to get into air from a moist soil or surface.—In reply, Dr. Edgeworth said that he did not accept the view that the cases of illness were due to leucomaines or ptomaines, for such a theory did not account for the case of enteric fever, nor for those of ulcerated throat, that had been mentioned in the discussion.

He thought that they did not arise from the sewer gas in the sewer, but from the excavated earth from around the old sewer, which presented most favourable conditions for multiplication and dissemination of micro-organisms it contained when brought to the surface and allowed to remain in the hot summer sun.

